# Influence of Social Media on Physician Selection and Medical Advice-Seeking Behaviors: A Cross-Sectional Study in Saudi Arabia

**DOI:** 10.7759/cureus.94973

**Published:** 2025-10-20

**Authors:** Motssim S Halawani, Badr Madani, Yazeed Alqurayqiri, Faisal Alsulami, Alhasan M Aljohani, Salem Alsulami, Khalid Alawlaqi

**Affiliations:** 1 Department of Family and Community Medicine, College of Medicine, University of Jeddah, Jeddah, SAU

**Keywords:** healthcare, health facts, health-seeking behavior, public health, social media

## Abstract

Background

Social media (SM) can help the public cultivate their autonomy by enriching the information offered by healthcare professionals (HCPs). It enables patients to obtain advice and complement offline information, perhaps increasing patient empowerment. The study aims to assess the influence of SM on the behavior of the general population when seeking healthcare advice and care from HCPs in Saudi Arabia.

Materials and methods

This was a cross-sectional study conducted in the Kingdom of Saudi Arabia, using a face-validated questionnaire by two experts and one statistician to receive participants' perceptions, attitudes, and experiences regarding SM in seeking medical care. Appropriate tests of significance and data presentation methods were used to explain the findings scientifically.

Results

Analysis of participant responses revealed that SM played a significant role in healthcare decision-making: its influence was rated as strong by 26% of participants and moderate by 47.8%. Snapchat, Telegram, and Instagram were the most commonly used platforms to access medical information from HCPs and medical institutions. Participants who belonged to the age groups of 35-44 and 45-54 years and those who were employed significantly showed a stronger influence than others (p<0.001). There was a statistically significant positive association observed between hours spent on SM and influence (p<0.05).

Conclusion

The impact of SM in seeking healthcare advice and care was found to be moderate among the participants. HCPs can establish interactive patient strategies using SM platforms. Physicians may reach out to potential patients and offer information about their healthcare organization or private practice using various SM platforms.

## Introduction

The term "social media (SM)" refers to a wide range of internet-based applications built on the ideological and technological foundations of Web 2.0, which are continually undergoing evolution [1]. These platforms facilitate the creation and exchange of user-generated content, enabling social interaction, collaboration, and the free exchange of information, ideas, and material among users in real-time [2-4]. For the purpose of this study, SM was conceptually defined as interactive, web-based platforms that facilitate the creation and exchange of user-generated content, community building, and real-time communication. This definition distinguishes SM from passive information retrieval via search engines (e.g., Google) or static health websites. The focus is on platforms that enable communal engagement and networking, such as Instagram, Snapchat, and X (formerly Twitter), where users interact with both healthcare professionals (HCPs) and peers to discuss, share, and seek health-related experiences and advice [1].

Despite the potential dangers, HCPs may take advantage of the opportunities presented by online resources to increase or enhance professional networking and education, organizational marketing, clinical services, patient education, and public health activities [2-8]. Patients and HCPs may be put at risk from the spread of false or misleading information, harm to the HCP's reputation, invasions of privacy, blurring of professional and personal roles, and legal complications [3,9-11]. Guidelines to prevent these dangers have been provided by a variety of healthcare establishments and professional organizations [9-13].

Over the past decade, there has been a dramatic rise in SM engagement among the public [14,15]. Since 2005, the percentage of American adults who use SM has grown rapidly from 7% to 65% in 2015 [16], with more recent estimates indicating continued growth to 73% of the total US population [17]. This trend is a global phenomenon; for instance, Singapore demonstrates one of the highest adoption rates, with 88.2% of its population using SM [18]. Globally, people of all ages and from all walks of life utilize SM. Moreover, nearly three billion individuals worldwide use Facebook, or more than one-third of the global population [17,18]. Additionally, YouTube has maintained its dominance as a video platform, with users watching billions of hours of content daily, while X (formerly Twitter) has an estimated 388 million monthly active users who generate a significant volume of daily posts. It is hardly a secret that resources like SM have a growing impact on a patient's choice of HCP [19,20].

Reviews are more likely to be favorable if patients are pleased with the treatment they received. However, unsatisfied patients will most likely provide a bad review. Maintaining a balance when responding to online reviews is vital, as they form an integral part of a patient acquisition strategy [21]. With more people doing their research online before committing to an HCP, patient ratings have become crucial [22]. Through psychographic segmentation, it becomes possible to recognize the kind of consumers most likely to depend on reviews and ratings. Patients can be categorized in terms of their lifestyles, motives, and values through a process called psychographic segmentation. Knowing a patient's segment allows HCPs to make educated guesses about them, such as their preferred mode of contact, their priorities in life, and the value they place on online reviews. SM serves as a valuable source of real-world evidence for HCPs, providing insights from patient interactions on symptoms and side effects that may be missed in clinical settings, thereby supporting more patient-centered care. It also enables the rapid dissemination of evidence-based knowledge among professionals, as demonstrated during the COVID-19 pandemic. However, this speed carries the risk of misinformation spread, necessitating rigorous vetting by HCPs. When used critically, SM can enhance interpersonal communication by offering a deeper context into patients' lives, fostering stronger therapeutic relationships and shared decision-making [23].

There is a wide range of viewpoints about the advantages, disadvantages, amount of information sharing, communication, and productivity obtained from the initial evidence of SM adoption in various healthcare practices. The effective incorporation of SM into healthcare practice can be better understood via research on patient characteristics, attitudes, and extrinsic factors that relate to usage purpose and frequency of use [24]. Saudi Arabia's healthcare system provides free universal care for citizens and public sector employees through government-funded facilities, while private sector employees and expatriates rely on mandatory employer-provided insurance. This structure directly ties healthcare access to employment and citizenship, making insurance status and income key determinants in individuals' ability to seek timely and preferred medical care, especially given reported challenges like long wait times in public facilities that may drive some toward private options requiring out-of-pocket payments [25,26].

To the best of our knowledge, no comprehensive studies have specifically explored public perception and practices regarding SM use for selecting HCPs in Saudi Arabia. However, global research confirms SM's significant role in healthcare decisions, with patients using it to access information on HCP credentials and reviews and a majority finding it useful for improving health understanding. This underscores a critical research gap in the Saudi context. Thus, this study aimed to explore the general public's perception, attitudes, and practices on SM in choosing HCPs for various healthcare services.

## Materials and methods

The authors developed a novel, self-administered questionnaire for this study. It was initially crafted in English to ensure it was grounded in the established terminology and constructs of the international literature on SM and healthcare. This approach facilitated the face validation process with experts and allowed for potential cross-cultural comparison. To ensure linguistic accuracy and cultural relevance for the Saudi Arabian population, the English version then underwent a standardized forward-backward translation process into Arabic.

To ensure the validity and reliability of the translated questionnaire, it was pilot-tested with a sample of 30 participants from the target demographic to evaluate its clarity, comprehensibility, cultural appropriateness, and average completion time. Based on the feedback received, a few adjustments were implemented, including simplifying language by replacing medical jargon with colloquial terms, rewording ambiguous questions to enhance neutrality and clarity, and improving formatting for better readability. These refinements ensured the final instrument was robust, culturally adapted, and easily understandable for the broader study population.

The option "subjective decision" was defined for participants as a personal choice based primarily on their own instincts, feelings, or initial impression, rather than on external factors such as recommendations from others or advertisements. The questionnaire was administered to a larger sample of participants through an online, self-administered format distributed via Google Forms. A cross-sectional analysis was conducted in the Kingdom of Saudi Arabia using a face-validated questionnaire by two experts and one statistician. Participants from all the provinces of Saudi Arabia were invited to take part in this population-based online survey.

Eligibility required participants to be aged ≥18 years, native Arabic speakers, and current residents of Saudi Arabia. While active SM use was not a formal inclusion criterion to maximize generalizability to the national population, where SM penetration is nearly 90%, the questionnaire incorporated internal validations to ensure analytical relevance. Detailed data on SM usage frequency, duration, and specific platforms were captured. This allowed for post-hoc stratification to analyze the primary outcomes specifically among active users (defined as daily use), ensuring the findings relevant to the research objectives are robust and derived from the appropriate subset of the sample. 

The required sample size was calculated using the formula for estimating a single population proportion with a specified absolute precision. Based on a review of existing literature, the assumed proportion (p) of the population influenced by SM in healthcare decisions was 0.5 (50%). This conservative estimate was selected to maximize the required sample size and ensure robustness, as it provides the maximum variability ($\text{p}\times\text{q}=0.25$). The calculation was performed for a 95% confidence level (Z=1.96) and a margin of error (d) of 2%. Applying the formula $\text{n}=\frac{\left(\text{Z}^{2}\times\text{p}\times\text{q}\right)}{\text{d}^{2}}$ yielded a minimum required sample size of 2401 participants. To account for potential non-response and incomplete submissions, the target sample size was increased by 15%, resulting in a final target of 2762 participants. The final collected sample of 2792 participants exceeded this target, confirming the adequacy of the sample for the study's analytical objectives.

A regional data collector from each of the five provinces was responsible for recruiting participants for the survey. The survey was open to all Saudi nationals and permanent residents who could participate during the study's time frame. Data were obtained from individuals who agreed to fill out the online pre-structured questionnaire after obtaining approval from the Bioethics Committee of Scientific and Medical Research of the University of Jeddah (approval number: UJ-REC-092). Before beginning the survey, informed consent was sought, and confidentiality was ensured. Study participants were protected in line with the World Medical Association's Declaration of Helsinki guidelines for research. An Arabic version of the questionnaires was distributed using Google Forms, which required a minimum of five minutes to complete. The data collection was conducted during the period of March 2022 to June 2022.

To ensure data integrity, duplicate responses were prevented by configuring the online survey to collect emails for technical validation while maintaining respondent anonymity. Recruitment via multiple regional data collectors across Saudi Arabia minimized selection bias. Social desirability bias was mitigated by emphasizing anonymity and the absence of right/wrong answers. Quantification of results involved descriptive statistics for demographic data, Likert scales (e.g., 1 (never) to 5 (always)) for practices/attitudes, and a composite "Influence Score" (summing four 5-point Likert items, range 4-20) for experiences. This score was categorized into Poor (≤49%), Moderate (50-74%), and Strong (≥75%) influence levels for analysis using IBM SPSS Statistics for Mac, Version 23.0 (IBM Corp., Armonk, New York, United States).

Regarding the quantification of results, the scoring system varied by section. The first section (sociodemographic details) contained categorical questions analyzed using frequencies and percentages. The second section (practices and attitudes) primarily utilized multiple-choice and Likert-scale questions. For example, questions on frequency of use were scored on a 5-point ordinal scale (e.g., 1 (never) to 5 (always)). The third section (experiences and influence) contained the core analytical measures. A key construct, "Influence of SM on seeking care", was measured using a series of four 5-point Likert statements (e.g., "SM influences my choice of a doctor", from 1 (strongly disagree) to 5 (strongly agree)). The scores for these four items were summed for each participant to create a composite "Influence Score" (theoretical range: 4-20). This continuous score was then categorized for analysis as follows: a score representing ≤49% of the total was classified as "Poor influence", 50-74% as "Moderate influence", and ≥75% as "Strong influence". All statistical analyses were performed using IBM SPSS Statistics for Mac, Version 23.0. The term "followed" was operationally defined for participants in the questionnaire to ensure consistent interpretation across different SM platforms. Specifically, participants were instructed that "following" a HCP or specialty.

Scoring system

The "Influence Score" was calculated as a composite measure from four, 5-point Likert-scale items (e.g., "SM influences my choice of a doctor", from 1 (strongly disagree) to 5 (strongly agree)). The scores for these four items were summed for each participant, yielding a theoretical range of 4-20. To justify this additive approach, the internal consistency of the four-item scale was assessed using Cronbach's alpha, which was found to be 0.89, indicating excellent reliability and confirming that the items measured a single, unified construct (the influence of SM).

Data management and statistical analysis

All the responses were downloaded and translated into the English language and then coded into a Microsoft Excel sheet (Microsoft Corporation, Redmond, Washington, United States). The coded responses were transferred to IBM SPSS Statistics for Mac, Version 23.0. An independent biostatistician was responsible for data analysis. All categorical variables were expressed in percentages and frequencies in tables and graphs as required. Pearson's chi-squared test was used to evaluate a possible association between categorical variables. A p-value of <0.05 was considered statistically significant at 95% CI.

To establish content validity, the content validity index (CVI) was calculated according to standard guidelines. A five-expert panel rated each item's relevance on a 4-point scale. The Item-CVI (I-CVI) represented the proportion of experts rating an item as quite/highly relevant (3 or 4), while the Scale-CVI (S-CVI) was calculated using both averaging (mean I-CVI) and universal agreement methods. Following psychometric standards, items required an I-CVI ≥0.78 for retention, and an S-CVI/Ave ≥0.90 indicated excellent overall validity [27].

The internal consistency of the questionnaire was assessed using Cronbach's alpha, adopting the widely accepted cut-off scores where a value of ≥0.7 is considered acceptable, ≥0.8 good, and ≥0.9 excellent. The questionnaire demonstrated high internal consistency, with an overall Cronbach's alpha of 0.89, confirming its good reliability before being administered to the larger study sample of 2792 participants.

**Figure 1:**
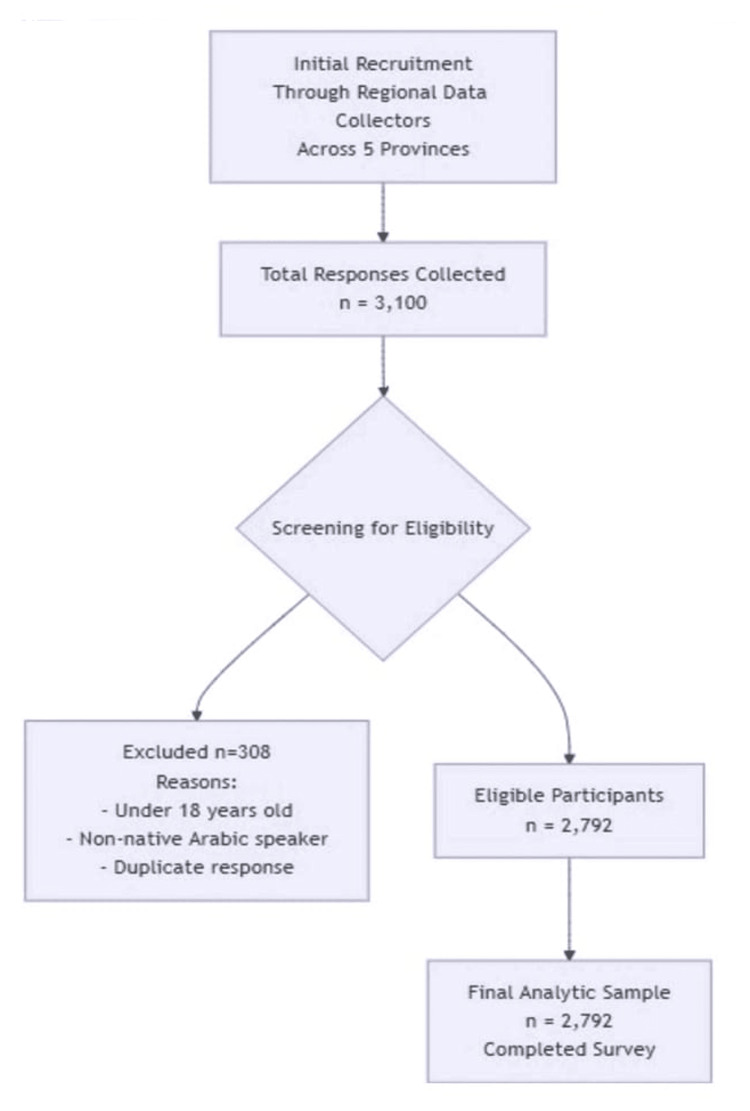
Study participant flow diagram

A participant flow diagram summarizing the recruitment process is presented in Figure 1. Initially, approximately 3100 responses were collected through an online survey distributed via regional data collectors across Saudi Arabia. Following the application of eligibility criteria, which required participants to be at least 18 years old, native Arabic speakers, and current residents of Saudi Arabia, 308 responses were excluded. The primary reasons for exclusion were being under the age of 18, identifying as a non-native Arabic speaker, or being identified as a duplicate submission through technical validation. The final analytic sample comprised 2792 eligible participants who completed the survey.

Ethical and legal considerations in recruitment

The study design and questionnaire were developed with consideration for the varying international regulations governing healthcare advertising. In certain jurisdictions, direct advertising of individual medical professionals is explicitly prohibited by law and medical ethics codes, allowing only for the dissemination of factual information (e.g., qualifications, office hours) through specific channels. This study focused on the organic behaviors of participants, namely, their voluntary actions to seek out and follow HCPs, rather than any direct advertising or solicitation by the researchers. All participant interactions with HCP SM content were self-directed. The questionnaire did not promote any specific HCPs and was designed solely to measure public perception and existing practices, thereby operating within ethical research boundaries across the studied regions.

## Results

We included 2792 participants who agreed to participate in the survey. The sociodemographic characteristics showed that 1004 (36%) belonged to the age group of 18-24 years, 1763 (63.1%) were females, a majority (90.5%) were Saudi citizens, 1548 (55.4%) were from the Western region, 1419 (50.8%) were married, 1925 (68.9%) had a university-level education, 1167 (41.8%) were employed, and 1425 (51%) had a monthly income of fewer than 5000 riyals (Table 1).

**Table 1:** Participants' sociodemographic data SAR: Saudi riyal

Variables		Number (n)	Percentage (%)
Age (years)	18-24	1004	36
	25-34	654	23.4
	35-44	582	20.8
	45-54	384	13.8
	≥55	168	6
Gender	Women	1763	63.1
	Men	1029	36.9
Nationality	Saudi	2527	90.5
	Non-Saudi	265	9.5
Province	Central region	316	11.3
	Eastern region	434	15.5
	Northern region	115	4.1
	Sothern region	379	13.6
	Western region	1548	55.4
Marital status	Unmarried	1264	45.3
	Married	1419	50.8
	Divorced	78	2.8
	Widow/widower	31	1.1
Educational qualification	Uneducated	4	0.1
	School level (elementary/intermediate/secondary)	665	23.8
	University (diploma/bachelor's degree)	1925	68.9
	Postgraduate (master's degree and above)	198	7.1
Employment	Employed	1167	41.8
	Not employed	721	25.8
	Business	114	4.1
	Student	790	28.3
Monthly income in SAR	<5000	1425	51
	5000-10000	604	21.6
	10001-20000	563	20.2
	20001-40000	145	5.2
	>40000	55	2

It was reported by more than half of the patients (50.2%) that they spend 2-5 hours per day on SM. The most commonly used SM platform is Snapchat (63.3%), followed by Telegram (45.4%), Instagram (45.2%), Twitter (41.9%), YouTube (38.6%), and TikTok (35.5%). About 35.3% reported that they always seek medical information using search engines, whereas 9% didn't seek any medical information. The majority of the participants believed that it was easier to reach doctors using SM platforms. It was reported by 1762 (63.1%) of the participants that they follow doctors on SM. The most commonly followed specialty on SM was found to be internal medicine (41.8%), followed by cosmetology (33%), dentistry (31.7%), psychiatry (26.7%), family medicine (26.4%), obstetrics and gynecology (23.5%), pediatrics (19.7%), surgery (18.8%), ophthalmology (12.6%), and ear, nose, and throat (ENT) surgery (10.3%).

When we explored factors that influence participants to select doctors, it was found that only 12.2% reported that advertisement on SM influences them to do so, whereas 59.5% mentioned it was the doctor's reputation. The finding that a doctor's reputation was the most influential factor (59.5%) for selecting an HCP underscores the enduring importance of trust and perceived competence. In the contemporary digital landscape, this reputation is increasingly built and gauged through SM platforms. Participants likely infer a doctor's reputation from a combination of aggregate metrics, such as overall star ratings and the volume of positive reviews, and qualitative assessments of the doctor's online persona, including the quality of informational content they share, their professionalism in engaging with patients online, and testimonials from other users. This suggests that while the underlying construct is traditional reputation, SM acts as a powerful and transparent conduit for its communication and evaluation.

When asked if SM platforms directly influenced their final selection of a specific doctor, 39.4% of participants reported no influence. Despite this, among the participants who were influenced, Snapchat, Twitter (X), and Instagram were the three most common platforms that impacted their decision-making process. More than half of the participants (50.4%) reported that they visited both private and governmental hospitals, whereas 23.3% visited only governmental hospitals. It was reported by 996 (35.7%) of the participants that they visited a doctor due to reviews, ratings, or recommendations they encountered on SM. Among these participants, the satisfaction level with their chosen doctor was high: about 33% were very satisfied, 40.4% were satisfied, 24.7% were somewhat satisfied, and only 1.9% were not satisfied at all (Table 2).

**Table 2:** Participants' practices regarding social media use The term "visit" in this context refers specifically to an outpatient consultation.

Variables		Number (n)	Percentage (%)
Time spent on social media (hours)	<2	401	14.4
	2-5	1402	50.2
	>5	989	35.4
Type of social media used	Snapchat	1767	63.3
	Instagram	1263	45.2
	Twitter	1170	41.9
	YouTube	1079	38.6
	TikTok	992	35.5
	WhatsApp	194	6.9
	Facebook	156	5.6
	Telegram	1268	45.4
	Google+	6	0.2
	Other	11	0.4
Obtaining medical information using search engines	Always	985	35.3
	Several times	1555	55.7
	Never	252	9
Social media makes it easier to approach doctors and ask questions	No	363	13
	Yes	2429	87
Following doctors on social media	No	1030	36.9
	Yes	1762	63.1
Specialty followed on social media (n=1762)	Internal medicine	736	41.8
	Surgery	332	18.8
	Family medicine	465	26.4
	Ear, nose, and throat (ENT) surgery	182	10.3
	Pediatrics	347	19.7
	Psychiatry	470	26.7
	Dentistry	558	31.7
	Obstetrics and gynecology	414	23.5
	Ophthalmology	222	12.6
	Cosmetology	582	33
	Others	24	1.36
Factors associated with doctor selection	Doctor's reputation	1661	59.5
	Subjective decision	1177	42.2
	Recommendation from others	1440	51.6
	Advertising on social media	342	12.2
	Appointment availability	12	0.43
	Others	24	0.86
Type of social media that had an impact on the decision to choose a therapist/doctor	Snapchat	657	23.5
	Twitter	620	22.2
	TikTok	188	6.7
	Instagram	587	21
	Facebook	48	1.7
	WhatsApp	14	0.5
	Other	379	13.6
	Not affected by any of the social media	1100	39.4
Have medical insurance	No	1761	63.1
	Yes	1031	36.9
Income influences the decision to see a therapist	Yes	1324	47.4
	Maybe	822	29.4
	No	646	23.1
Type of hospital to visit for medical care	Government	651	23.3
	Private	735	26.3
	Both	1406	50.4
Visited a doctor based on the feedback seen on social media	No	1796	64.3
	Yes	996	35.7
Satisfaction after visiting a doctor (n=966)	Not satisfied	19	1.9
	Somewhat satisfied	246	24.7
	Satisfied	402	40.4
	Very satisfied	329	33

**Figure 2:**
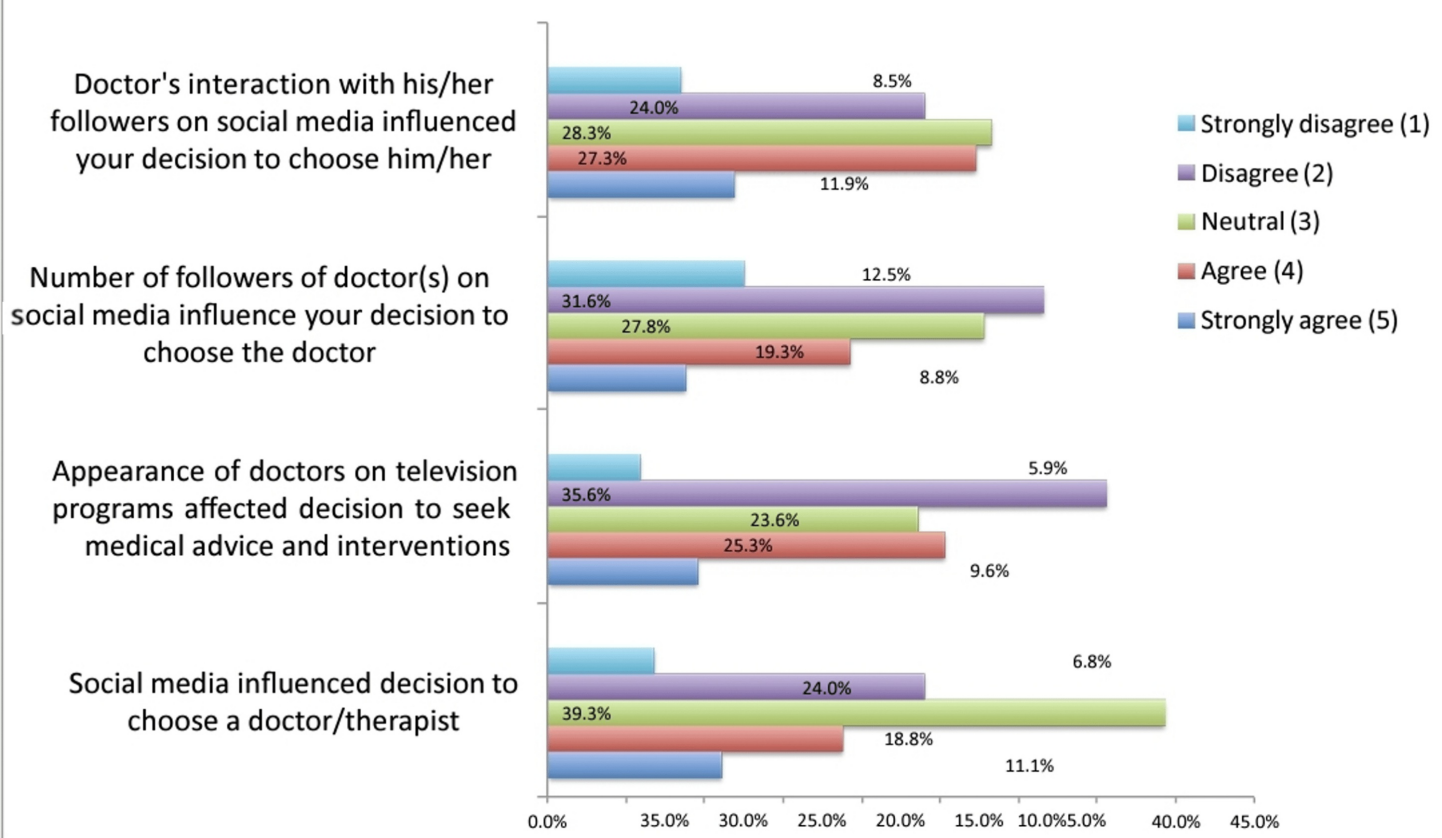
Five-point Likert scale to rate the influence of social media on participants' experiences with doctors

We used a five-point Likert scale to rate the influence of SM on participants' experiences with doctors (Figure 2). About 29.9% agreed that SM had influenced their decision to choose a therapist or doctor. The appearance of doctors on television-based SM platforms, such as professional YouTube channels, Instagram Live sessions, or televised interviews that were later clipped and shared on platforms like Twitter (X) or Facebook, influenced 34.9% of participants to seek medical advice and interventions from them. About 34.1% of them didn't believe that the number of doctors' followers on SM influenced them to seek care from the doctor. On the other hand, about 39.2% agreed that attending doctors' interactions with his/her followers on SM influence their decision to choose the doctor (Figure 2).

Psychometric properties

Prior to analyzing the primary outcomes, the psychometric properties of the four-item "Influence Scale" were evaluated. The scale demonstrated high internal consistency, with a Cronbach's alpha of 0.89, confirming its reliability. Content validity was established during the development phase through expert review, ensuring the items were relevant and comprehensive in capturing the construct of SM influence. The resulting composite score was categorized for interpretability as follows: scores representing ≤49% of the total (≤9.8) were classified as "Poor influence", 50-74% (10-14.8) as "Moderate influence", and ≥75% (≥15) as "Strong influence". We evaluated the relationship of various participants' characteristics with the SM influence level (Table 3).

**Table 3:** Relationship between SM influence and various sociodemographic characteristics of the participants ^1^Chi-squared test; *: statistically significant at p<0.05. SM: social media

Variables		SM influence n (%)			P-value^1^
		High	Moderate	Low	
Age (years)	18-24	239 (23.8%)	445 (44.3%)	320 (31.9%)	<0.001^*^
	25-34	165 (25.2%)	329 (50.3%)	160 (24.5%)	
	35-44	173 (29.7%)	275 (47.3%)	134 (23%)	
	45-54	121 (31.5%)	195 (50.8%)	68 (17.7%)	
	≥55	27 (16.1%)	90 (53.6%)	51 (30.4%)	
Gender	Women	458 (26%)	860 (48.8%)	445 (25.2%)	0.238
	Men	267 (25.9%)	474 (46.1%)	288 (28%)	
Nationality	Saudi	668 (26.4%)	1191 (47.1%)	668 (26.4%)	0.086
	Non-Saudi	57 (21.5%)	143 (54%)	65 (24.5%)	
Province	Central region	64 (20.3%)	149 (47.2%)	103 (32.6%)	<0.001^*^
	Eastern region	110 (25.3%)	220 (50.7%)	104 (24%)	
	Northern region	39 (33.9%)	46 (40%)	30 (26.1%)	
	Southern region	126 (33.2%)	174 (45.9%)	7 (20.8%)	
	Western region	386 (24.9%)	745 (48.1%)	417 (26.9%)	
Marital status	Unmarried	295 (23.3%)	576 (45.6%)	393 (31.1%)	<0.001^*^
	Married	397 (28%)	704 (49.6%)	318 (22.4%)	
	Divorced	27 (34.6%)	36 (46.2%)	15 (19.2%)	
	Widow	6 (19.4%)	18 (58.1%)	7 (22.6%)	
Educational level	Uneducated	3 (75%)	0 (0%)	1 (25%)	0.335
	School level	174 (26.2%)	314 (47.2%)	177 (26.6%)	
	University	503 (26.1%)	921 (47.8%)	501 (26%)	
	Postgraduate and above	45 (22.7%)	99 (50%)	54 (27.3%)	
Employment	Employed	341 (29.2%)	553 (47.4%)	273 (23.4%)	<0.001^*^
	Not employed	184 (25.5%)	365 (50.6%)	172 (23.9%)	
	Business	29 (25.4%)	62 (54.4%)	23 (20.2%)	
	Student	171 (21.6%)	354 (44.8%)	265 (33.5%)	
Monthly income	<5000	340 (23.9%)	682 (47.9%)	403 (28.3%)	0.037^*^
	5000-10000	181 (30%)	284 (47%)	139 (23%)	
	10000-20000	159 (28.2%)	270 (48%)	134 (23.8%)	
	20000-40000	29 (20%)	74 (51%)	42 (29%)	
	>40000	16 (29.1%)	24 (43.6%)	15 (27.3%)	
Time spent on SM (hours)	<2	88 (21.9%)	186 (46.4%)	127 (31.7%)	0.025^*^
	2-5	366 (26.1%)	693 (49.4%)	343 (24.5%)	
	>5	271 (27.4%)	455 (46%)	263 (26.6%)	

It was found that participants belonging to the age groups of 35-44 and 45-54 years had demonstrated a significantly stronger influence than other age groups (p<0.001). Participants from the southern province significantly showed strong influence, and those from the central province demonstrated comparatively low influence (p<0.001). Unmarried participants demonstrated a significantly poorer influence, whereas strong influence was comparatively higher among married participants (p<0.001). Participants who were employed had a significantly stronger influence than others (p<0.001), and those who had less salary (<5000 riyals) demonstrated a significantly poorer influence than others (p=0.037). There was a positive association found between the time hours spent on SM and influence, where participants who used SM for more than five hours had comparatively demonstrated a stronger influence than others who spent less time (p=0.025).

## Discussion

The implications of patients using SM for health-related purposes vary, posing both opportunities and threats. Since an increasing number of the population are turning to SM for health-related motives, HCPs will need to consider both the purported benefits and the possible drawbacks of patients' increasing reliance on online health communities [28]. This behavior aligns with the broader trend of patients turning to SM for convenient access to information [29] and to supplement offline information [30], which may lead to improved patient engagement [31]. In particular, online health information has been shown to increase patients' confidence in managing chronic conditions, as they can cross-reference medical advice, clarify doubts, and prepare questions for subsequent consultations [32]. It is crucial to establish trustworthy online communication channels to avoid the exacerbation of health issues since anybody with access to SM may provide information or advice on how to cope with a specific health issue [33].

Because of this, healthcare institutions and medical facilities have begun actively investigating the role that SM plays in the relationship that exists between patients and HCPs, as well as how this aspect of the interaction correlates to various health informatics systems. In our study, we asked the participants about the many ways in which they use SM and what motivates them to do so. After conducting an analysis of the practices and experiences, it has become abundantly evident that the majority of people do not use SM platforms to circumvent HCPs; rather, they use these channels as a supplement to the services provided by HCPs in order to meet their health information and peer support needs. This behavior aligns with the broader trend of patients turning to SM for convenient access to information, emotional support, and community connection, which complements rather than replaces formal medical advice [34-36].

People have the perception that their relationship with their HCPs is a more clinical one, in which HCPs give professional advice about the problem and propose treatments based on their medical expertise [37]. Our findings suggest that the SM significantly influences the decision to seek medical attention for over half of the participants. The forms of SM usage described by this study pertain to the manner in which people utilize SM to fulfill unmet health needs. Snapchat, Twitter, and Instagram were the commonly used SM for healthcare purposes. In Saudi Arabia, WhatsApp, Twitter, Instagram, and Snapchat are some of the commonly used SM platforms to acquire health information [38]. Snapchat is one of the popular SM platforms used in Saudi Arabia, and its use is found to be significantly higher among the younger age group [39]. Our finding is not in agreement with some previous studies [40,41] done in the region that had reported WhatsApp as the most frequently used SM for health information. Our study found a significant difference in levels of SM influence based on multiple demographic factors. Participants' age, employment status, income, and time spent on SM were strong predictors of its influence. Furthermore, marital status and geographic province also demonstrated significant associations.

The variation by province is likely attributable to differing levels of healthcare infrastructure, access to specialists, and regional digital literacy campaigns, which may influence reliance on SM for healthcare information. The significant association with marital status may be explained by the different health information-seeking behaviors and social support networks available to married versus unmarried individuals; for instance, unmarried participants may rely more heavily on digital communities for health-related advice and support. These findings underscore that the influence of SM on healthcare decisions is not uniform but is instead shaped by a complex interplay of socioeconomic, geographic, and personal social factors. However, there were no differences found in the SM influence based on the educational level.

This is in contrast to the findings of another study done by AlMuammar et al., which reported that higher education was found to use SM for healthcare advice and information than people with lower educational levels [42]. Another challenge for HCPs in using SM is that they often have limited time to interact with the public [43]. Appointments with primary care physicians and other specialists are frequently scheduled in 15-30-minute sessions throughout the day. They see walk-ins and those who schedule appointments at the last minute when they have openings in their schedule. This makes it difficult for physicians to maintain a regular posting schedule on SM. People who are concerned about their health and who use SM often may be interested in following physicians or other medical experts who share regular updates [44]. Despite the many limitations, SM can improve an HCP's capacity to provide excellent care for their patients. The professional dynamic between patients and the healthcare system as a whole may be dramatically enhanced by SM's capacity to engage directly with HCPs.

One of the strengths of our study is the larger sample and the participants' heterogeneity observed in sociodemographic characteristics. Thus, the participants' attitudes and behavior in this study could be generalizable to the majority of the Saudi population to some extent. Our study also has some limitations. Firstly, we used a self-administered online questionnaire to collect information. Therefore, the possibility of self-report bias and social desirability bias cannot be missed. Secondly, we attempted to distribute the survey impartially through their professional and personal social networks; however, there is a difference in internet and other SM usage based on age, gender, and other demographic characteristics, which may have limited the survey's reach and affected the generalizability of the findings.

Study limitations

This study is subject to several limitations. The cross-sectional design precludes causal inferences, only permitting the identification of associations. Reliance on an online, self-administered survey introduces the potential for selection bias, particularly an underrepresentation of groups with lower digital literacy, and self-reporting biases such as recall and social desirability. The generalizability of the findings may be impacted by the overrepresentation of participants from the western province and females. Finally, while data on insurance and income were captured, the structure of Saudi Arabia's healthcare system, which directly ties access to employment and citizenship, remains a potential confounding factor. The reliance on and perception of SM for selecting HCPs may differ significantly between citizens with guaranteed public healthcare and those reliant on private insurance, a nuanced interplay that warrants specific investigation in future research.

## Conclusions

The findings of this study showed that the influence that SM had on seeking healthcare and advice from physicians and other HCPs was found to be moderate to high. Snapchat, Instagram, and Twitter were the commonly used platforms to follow HCPs. There was a strong positive association observed between time spent on SM and the influence made on seeking healthcare. As the usage of SM grows more widespread, it is increasingly being used in healthcare settings. It is imperative that medical experts keep exploring new ways to put these technologies to work in the service of improving patient care.
